# Assessing the impact of the *Dobbs v. Jackson* decision on abortion attitudes by abortion identity labels: a mixed-methods longitudinal study

**DOI:** 10.1080/26410397.2025.2518669

**Published:** 2025-06-16

**Authors:** Xiana Bueno, Lucrecia Mena-Meléndez, Brandon L. Crawford, Ronna C. Turner, Wen-Juo Lo, Kristen N. Jozkowski

**Affiliations:** a Associate Research Scientist, Department of Applied Health Science, School of Public Health, Indiana University, Bloomington, IN, USA; b Assistant Research Scientist, Department of Applied Health Science, School of Public Health, Indiana University, Bloomington, IN, USA; c Associate Professor, Department of Applied Health Science, School of Public Health, Indiana University, Bloomington, IN, USA; d Professor, Educational Statistics and Research Methods, College of Education and Health Professions, University of Arkansas, Fayetteville, AR, USA; e Associate Professor, Educational Statistics and Research Methods, College of Education and Health Professions, University of Arkansas, Fayetteville, AR, USA; f William L. Yarber Endowed Professor in Sexual Health, Department of Applied Health Science, School of Public Health, Indiana University, Bloomington, IN, USA.

**Keywords:** abortion attitudes, abortion identity, attitudinal change, longitudinal survey, public opinion, *Dobbs v. Jackson Women's Health Organization*

## Abstract

Landmark legislative events can shift public opinion. We conducted a longitudinal survey examining abortion attitudes before and after *Dobbs v. Jackson* which overturned *Roe v. Wade* in 2022. Wave 1 (*N* = 1,014) was conducted in June 2022, and Wave 2 (*N* = 792) in October–November 2022. Using bivariate analyses, we assessed people’s attitudes towards the Dobbs decision and potential changes in abortion attitudes over time, across different abortion identity sub-groups (e.g. pro-life, pro-choice). Results indicate that people were informed about (90%) and disagreed (56%) with the decision, and did not report or experience a change in attitudes after the decision (68–73%). However, among those who did change, respondents were more inclined to endorse legal abortion after the decision (19–22%) than indicate abortion should not be legal (6–13%). Through analysing open-ended data, we found that participants more inclined to endorse legal abortion described the ruling as eroding personal rights, government intrusion, and threatening access to healthcare. Participants less inclined to endorse legal abortion indicated the ruling reinforced their belief in defending fetal rights. While not necessarily advocating outright illegality, such participants favoured stricter regulations. Notably, people who identified as “both/neither/prefer not to answer” tended to disagree with the Dobbs decision and lean towards greater endorsement of legal abortion. Uncertainty regarding (dis)agreement with the Dobbs decision was also higher among people who identified as pro-life and “both/neither/prefer not to answer” than among those who identified as pro-choice. These findings highlight important nuances that exist in abortion attitudes beyond the perceived dichotomy of the pro-life/pro-choice spectrum.

## Plain language summary

In 2022, the United States Supreme Court made a significant decision in the *Dobbs v. Jackson* case, overturning the earlier *Roe v. Wade* ruling. This change affected the legal status of abortion across the country. Our study looked at how people’s views on abortion changed before and after this decision. We conducted a survey over time, asking people about their opinions on the *Dobbs v. Jackson* decision and whether their attitudes towards abortion had changed. Using bivariate survey analysis, we found that most people in our study knew about the decision and disagreed with it. For the majority, their views on abortion did not change after the decision. Yet, for those whose views changed, more people endorsed legal abortion than opposed it post-Dobbs. Through analysing open-ended data, we found that people who reported more endorsement of legal abortion thought the decision took away individual rights and threatened healthcare access. Those who reported lower endorsement of legal abortion indicated the decision helped protect fetal rights. We found that people who did not fully identify as pro-life or pro-choice – those who identified as “both, neither, or prefer not to answer” – were more likely to disagree with the *Dobbs v. Jackson* decision and endorse legal abortion. Additionally, those who identified as “both, neither, or prefer not to answer” were more uncertain regarding whether they agreed with the decision. Our findings suggest that people’s views on abortion are more complex than pro-life or pro-choice labels suggest. Variation in views regarding abortion do not fit neatly into these two categories.

## Résumé

Des événements législatifs marquants peuvent influencer l’opinion publique. Nous avons réalisé une enquête longitudinale examinant les attitudes à l’égard de l’avortement avant et après l’arrêt *Dobbs vs Jackson*, qui a infirmé l’arrêt *Roe v. Wade* en 2022. La première vague (*n* = 1014) a été menée en juin 2022, et la deuxième vague (*n *= 792) en octobre-novembre 2022. À l’aide d’analyses bivariées, nous avons évalué l’attitude des personnes à l’égard de la décision Dobbs et les changements potentiels dans les attitudes envers l’avortement au fil du temps, dans plusieurs sous-groupes d’identité en matière d’avortement (par exemple pro-vie, pro-choix). Les résultats indiquent que les personnes connaissaient la décision (90%) et la désapprouvaient (55,7%) et qu’elles n’ont pas signalé ni expérimenté de changement d’attitude après la décision (68–73%). Néanmoins, parmi celles qui ont changé, les répondants étaient plus enclins à approuver l’avortement légal après la décision (19–22%) qu’à indiquer que l’avortement ne devrait pas être légal (6–13%). En analysant des données ouvertes, nous avons constaté que les participants les plus enclins à approuver l’avortement légal décrivaient l’arrêt comme une atteinte aux droits individuels, une ingérence de l’État et une menace pour l’accès aux soins de santé. Les participants moins enclins à soutenir l’avortement légal ont indiqué que la décision renforçait leur volonté de défendre les droits du fœtus. Sans nécessairement plaider pour une totale illégalité, ces participants étaient favorables à une réglementations plus stricte. Il est à noter que les personnes s’identifiant comme « les deux/ni l’un ni l’autre/préfère ne pas répondre » tendaient à être en désaccord avec décision Dobbs et à pencher davantage pour un soutien plus marqué à l’avortement légal. L’incertitude concernant l’accord (ou le désaccord) avec la décision Dobbs était également plus élevée chez les personnes s’identifiant comme pro-vie et « les deux/ni l’un ni l’autre/préfère ne pas répondre » que parmi celles qui s’identifiaient comme pro-choix. Ces résultats mettent en évidence d’importantes nuances qui existent dans les attitudes à l’égard de l’avortement, au-delà de la dichotomie perçue entre pro-vie et pro-choix.

## Resumen

Los acontecimientos legislativos trascendentales pueden cambiar la opinión pública. Realizamos una encuesta longitudinal para examinar las actitudes relativas al aborto antes y después de *Dobbs c. Jackson,* que revocó *Roe c. Wade* en 2022. La Ronda 1 (*N* = 1,014) se realizó en junio de 2022 y la Ronda 2 (*N* = 792) entre octubre y noviembre de 2022. Utilizando análisis bivariados, evaluamos las actitudes de las personas hacia la decisión del caso Dobbs y los posibles cambios en las actitudes relativas al aborto con el paso del tiempo, entre diferentes subgrupos de identidad respecto al aborto (ej., pro-vida, pro-elección). Los resultados indican que las personas estaban informadas sobre (90%) y estaban en desacuerdo con (55.7%) la decisión, y no notificaron ni tuvieron un cambio de actitud después de la decisión (68–73%). Sin embargo, entre las que sí cambiaron, las personas encuestadas estaban más inclinadas a aprobar la interrupción legal del embarazo (ILE) después de la decisión (19–22%) que a indicar que el aborto no debe ser legal (6–13%). Al analizar datos de preguntas abiertas, encontramos que las personas encuestadas que estaban más inclinadas a aprobar la ILE describieron la sentencia como una erosión de los derechos personales, una intrusión del gobierno y una amenaza contra el acceso a los servicios de salud. Las personas encuestadas menos inclinadas a aprobar la ILE indicaron que la sentencia reafirmaba su creencia en defender los derechos del feto. Aunque no necesariamente abogaron por la ilegalidad absoluta, tales participantes favorecieron normativas más estrictas. Notablemente, las personas que se identificaron como “ambas/ninguna de las dos/prefiero no responder” tendían a estar en desacuerdo con la decisión del caso Dobbs y a inclinarse hacia una mayor aprobación de la ILE. La incertidumbre sobre el (des)acuerdo con la decisión del caso Dobbs también fue mayor entre las personas que se identificaron como provida y como “ambas/ninguna de las dos/prefiero no responder” que entre aquéllas que se identificaron como pro-elección. Estos hallazgos ponen de relieve importantes matices que existen en las actitudes hacia el aborto más allá de la dicotomía percibida del espectro provida o pro-elección.

## Introduction

On June 24, 2022, the Supreme Court of the United States’ (SCOTUS) decision in *Dobbs v. Jackson Women’s Health Organization* (i.e. *Dobbs v. Jackson*) overturned *Roe v. Wade*, the 1973 Supreme Court decision that, for almost fifty years, indicated that abortion up to viability was a constitutional right.^1^ Landmarked legislative events of this magnitude do not generally go unnoticed but rather have the potential to strengthen people’s opinions or prompt attitudinal changes in public opinion.^2^ As such, we examined people’s perceptions of whether their attitudes towards legal abortion changed after the *Dobbs* decision. Additionally, one might expect that after the *Dobbs* decision, people who identify as pro-life would show lower endorsement for legal abortion and people who identify as pro-choice would show greater endorsement of legal abortion. However, these labels may not fully account for the complexity and multidimensionality that exist in people’s attitudes and beliefs regarding abortion.^3–5^ To further examine the change in people’s attitudes towards abortion post-*Dobbs*, we compared attitudes towards abortion legality pre- and post-*Dobbs* decision, accounting for abortion labels. Such findings are important for and relevant to researchers, media, and policymakers interested in understanding how the *Dobbs v. Jackson* decision shaped public attitudes towards abortion as well as those of healthcare professionals and advocacy groups, as they shed light on the social and political climate surrounding abortion which may inform medical practices, public health strategies, and advocacy efforts.

### Attitudinal changes towards abortion legality and the role of the Supreme Court

Literature exploring whether and how people’s attitudes towards social issues change is abundant. Some scholars suggest that social attitudes are shaped during young adulthood, laying a foundational role in a person’s value system that often persists over time.^6–8^ However, not all attitudes are rigid. Some attitudes demonstrate stability, and others show potential for change. For instance, the *settled disposition model* of attitudinal change posits that some attitudes formed earlier in life play a key role in people’s value systems and tend to endure over time.^9,10^ Baumgarten et al.^11^ found that this is particularly true regarding abortion attitudes since they can be rooted in morality and closely linked to religious beliefs. In contrast, drawing on the *active updating model*, Kiley and Vaisey^9^ argued that people can and do modify their attitudes on social issues in response to new experiences, discourses, and information. This model underscores the role played by education in exposing people to diverse perspectives and information, which in turn may foster attitudinal changes.^12^

More specifically, several studies have explored the influence of Supreme Court rulings on public opinion and potential attitude shifts. Dahl^13^ theorised that Supreme Court rulings are generally accepted by the public with minimal subsequent controversy; this expectation was later named the *positive response hypothesis*.^14^ While their research did indeed identify an increase in support for abortion following the decision in *Roe v. Wade*, it also highlighted a growing polarisation in attitudes towards abortion. And in the decades since *Roe v. Wade* was decided, consensus and acceptance regarding abortion had not been reached. In contrast, according to the *elaboration likelihood model*,^15^ once people conform to an opinion on a particular issue, further analysis of the issue is unlikely to alter their initially formed opinion. Drawing on the elaboration likelihood model, Johnson and Martin^16^ argued that for many people, the Supreme Court holds a high degree of legitimacy and credibility. However, for others with a clear stance on an issue, subsequent Supreme Court rulings on the same issue, even if they contradict people’s formed opinion, are unlikely to significantly alter their opinion. Thus, according to this theoretical framework, it is possible that the impact of the *Dobbs* decision on public attitudes towards abortion would be limited.

### The use of pro-life and pro-choice labels in abortion attitudes research

The terms pro-life and pro-choice emerged in the context of the abortion debate in the United States during the 1960s and 1970s.^17^ The term pro-life gained projection as a label representing individuals and groups who opposed abortion and emphasised the belief in the sanctity of human life beginning *in utero* and the protection of the “unborn child”.^18^ Conversely, the term pro-choice emerged as a label for individuals and groups advocating for the legal right of women to choose whether to have an abortion by emphasising the importance of women’s reproductive rights and bodily autonomy.^19^ Additionally, the term reproductive justice emerged more recently^20^ as a socio-political term; however, it has not replaced pro-choice in abortion discourse. The pro-life and pro-choice labels are deeply entrenched in the public discourse surrounding abortion and have been widely used by activists, policymakers, and researchers as polarising terms describing opposing views on abortion.^21^ In public opinion research on abortion, these terms have been instrumental in framing and understanding people’s attitudes and beliefs regarding abortion legislation and restrictions. Many polling organisations, such as Gallup, Pew Research Center, and the Kaiser Family Foundation, use these labels to categorise respondents based on their stance on abortion.^22^ Likewise, scholars in academic research also use these terms, sometimes conflating them with attitudes, as well as analysing trends in attitudes and demographic differences among those who endorse or do not endorse legal abortion.^17,23^ Despite their widespread use, it is essential to recognise that these labels can oversimplify the complexity of people’s beliefs about abortion.^3^ Many people hold nuanced views that may not neatly align with these binary labels, or in a dichotomy more generally; in fact, many people report identifying with both labels.^22^ Additionally, there is variability in how people conceptualise and define these terms.^24^ For example, these terms are US-centric, and foreign-born Spanish-speaking populations in the US may interpret terms differently than those who are US-born.^25^

Examining how public opinion on legal abortion is shaped by key Supreme Court rulings, such as the *Dobbs v. Jackson* decision, is crucial because public sentiment can influence policy, healthcare access, and societal attitudes, which have further implications for fundamental rights. At the legislative level, while Supreme Court rulings establish legal precedent, public opinion can drive legislative responses. Thus, greater public support for abortion rights may prompt lawmakers to introduce protective policies, whereas opposition could lead to further restrictions. These legislative shifts can have direct ramifications on healthcare availability and access, affecting people’s ability to obtain necessary reproductive care. Additionally, public opinion on abortion is strongly tied to people’s perception of abortion acceptability and stigma.

### Aim and purpose

Despite the importance of *Dobbs*, there is limited research on its impact on people’s opinions or attitudes. To fill this gap, we used longitudinal data to assess (1) people’s perceptions of attitudinal change towards abortion legality after *Dobbs* and (2) potential changes in people’s attitudes towards abortion legality, measured before and after the *Dobbs* decision, accounting for abortion labels. Additionally, using open-ended data, we also assessed the reasons participants provided for their attitudinal changes post-*Dobbs*. Specifically, we explored the underlying reasons people provided for their perceived greater or lower endorsement of legal abortion following the *Dobbs* decision.

We pursued four main objectives. Firstly, we examined people’s awareness and agreement with the *Dobbs v. Jackson* decision after the decision. Secondly, we assessed whether participants’ abortion views changed over time by evaluating two measures of change: (1) a perceived measure of attitudinal change and (2) a measure of response change on a survey item on attitudes towards abortion legality asked before and after the *Dobbs* decision. Thirdly, we assessed participants’ rationale for attitudinal change by analysing open-ended survey data. Fourthly, using multinomial regression models, and after controlling for common sociodemographic predictors of abortion attitudes, we assessed whether identification with abortion identity labels predicts greater or lower endorsement of legal abortion after the *Dobbs v. Jackson* decision.

## Data and methods

### Study design

In 2022, we launched an online longitudinal survey using Ipsos's probability-based KnowledgePanel®, a web-based panel designed to be representative of the US household population. Ipsos uses address-based sampling (ABS) to develop its KnowledgePanel^®^, which is representative of US households. To accommodate those without internet access or devices, Ipsos provides internet-enabled devices. Ipsos’ KnowledgePanel® has been widely used in academic and policy research as a nationally representative source of public opinion in the United States.^26^ The survey assessed US adults’ awareness, knowledge, and attitudes regarding abortion laws before and after the SCOTUS *Dobbs v. Jackson* decision announcement. We launched Wave 1 (*N* = 1,014) of the survey in June 2022, before the decision announcement. Most of the sample (93.2%) completed the survey before the decision announcement on June 24 at 10:00 am EST.

Wave 2 was fielded with the same participants from Wave 1, between October 17 and November 11, 2022 (*N* = 792). The attrition rate was 21.9%. In the present study, we included all respondents who responded to both surveys. To increase the validity of our research question and design, we excluded participants (*n* = 58) who completed Wave 1 after June 24, 2022, at 10:00 am EST – the day and time when the decision was announced. Thus, our final analytical sample comprised 734 participants.

We administered our survey in English and Spanish, with approximately 87.7% of respondents answering in English (*n* = 644) and 12.3% of respondents answering in Spanish (*n* = 90). English and Spanish are the first and second most commonly spoken languages in the US, respectively. Additionally, the Latinx/Hispanic population is the largest minority group in the US, representing 19.5% of the population in 2023 according to the US Census.^27^ As a result, collecting public opinion data in Spanish, in addition to English, is increasingly important. The multilanguage design of the survey items followed a parallel translation development approach, with English as the source language. The translation process adhered to the Translate, Review, Adjudicate, Pretest, and Document (TRAPD) framework.^28^ This approach entailed a team-based translation process with several iterations of review and reconciliation in both languages. The survey instrument was initially developed by our monolingual and bilingual research team in English (as the source document) while considering item wording in Spanish. This initial instrument was then translated into Spanish by the first two authors, who are bilingual (English/Spanish) and native Spanish speakers. Modifications to the source (i.e. English) items were made when a more adequate translation from the target language (i.e. Spanish) was required. Refinement of the instrument was made in both languages via an iterative process as recommended by the TRAPD framework. (Please see Table A2 in the Appendix for a complete list of English and Spanish survey questions and response options analysed in this study.)

Weights were calculated using an iterative proportional fitting with gender, age, education, household income, race/ethnicity, home ownership, household size, metropolitan area, language dominance, and region of the US. Weights aligned with the US Census Bureau’s American Community Survey (ACS) and Current Population Survey (CPS). Materials and study protocols for both waves were approved by the Institutional Review Board (IRB) at Indiana University, providing ethics approval prior to data collection on 21 February 2022 (IRB # 14136). Participants viewed an informed consent page before starting the survey and had to actively agree before proceeding. This process aligns with ethical standards for online survey research and ensures informed consent. We obtained informed consent from all participants before beginning each survey. Participants received an incentive through the panel administration points system, which can later be redeemed for gift cards, which is standard practice for Ipsos’ KnowledgePanel® and other survey aggregators.

### Measures

*Awareness and Support of the Decision to Overturn *Roe v. Wade* (Wave 2)*.

After providing a statement of information regarding *Roe v. Wade*,* we asked (a) “Before taking this survey, had you heard that *Roe v. Wade* was overturned (gotten rid of) in June 2022?”, with response options “Yes” and “No,” and (b) “Do you agree or disagree with the decision to overturn (get rid of) *Roe v. Wade*?”, with a 5-point Likert response option: “Strongly agree,” “Agree,” “Unsure,” “Disagree,” and “Strongly disagree.”

#### Changes in attitudes towards abortion after *Dobbs v. Jackson* (Wave 1 and Wave 2)

We used two different measures of attitude change. Firstly, a perceived measure of change in attitudes towards abortion based on self-reported sentiment using Wave 2 data. Specifically, we asked participants, “Have your opinions about the legality of abortion changed after *Roe v. Wade* was overturned?” with response options: “No, my opinions on the legality of abortion have not changed,” “Yes, now I am more supportive of abortion,” and “Yes, now I am more opposed to abortion.” We then asked an open-ended follow-up question to those who reported a change in views, asking them to explain why their views changed. We asked, “Can you explain why you have become [more supportive]/[more opposed] to abortion being legal after *Roe v. Wade* was overturned (gotten rid of)?” Secondly, we measured response change across waves by comparing the responses to the same survey item – “Do you think abortion should be legal in all cases, legal in most cases, illegal in most cases, or illegal in all cases?” – in both waves. Based on participants’ responses from Wave 1 and Wave 2, we coded the direction of attitudinal change (i.e. greater endorsement, no change, lower endorsement). For example, if a participant answered “illegal in most cases” in Wave 1 and “legal in most cases” in Wave 2, we coded this as a greater endorsement of legal abortion. Similarly, if a participant responded “legal in most cases” in Wave 1 and “legal in all cases” in Wave 2, we also coded this as a greater endorsement of legal abortion.

#### Abortion identity

We also assessed how respondents self-identified with abortion identity labels using a 9-point scale measure. Response options included: “completely pro-choice,” “moderately pro-choice,” “slightly pro-choice,” “both pro-choice and pro-life,” “slightly pro-life,” “moderately pro-life,” “completely pro-life,” “neither pro-choice nor pro-life,” and “prefer not to answer.” We collapsed responses into three categories: (1) pro-choice; (2) both/neither/prefer not to answer; and (3) pro-life.

#### Sociodemographic characteristics

We controlled for other sociodemographic characteristics: gender (man/woman), age, primary race/ethnicity (White/Latinx/Black-African American/Multiracial-Other), education (high school or less/some college/bachelor or higher), church attendance (weekly-monthly/yearly/never), party identification (Republican/Democrat/Other-Any), and region of residence (Northeast/Midwest/South/West). A description of our sample’s demographic characteristics can be found in Table A1 in the Appendix.

### Analysis

We used a mixed methods approach to capture both the broad patterns and the deeper, contextual factors shaping abortion attitudes after *Dobbs v. Jackson*. Quantitative data identified trends of change, while qualitative insights revealed the reasoning behind change, providing a more comprehensive understanding than either method alone.

#### Bivariate analysis

We first explored bivariate combinations of abortion law awareness and agreement/disagreement based on how people identified with the abortion identity labels. We provided descriptive-level findings – frequencies and weighted percentages – of our variables of interest. We also conducted chi-square tests of association to examine whether there were positive associations between abortion law awareness and agreement/disagreement (Wave 2 data only) and abortion identity labels.

#### Open-ended data analysis

We analysed open-ended responses from participants who reported that the *Dobbs v. Jackson* decision changed their views towards legal abortion, either towards lower or greater endorsement of legal abortion. The first two authors, both bilingual and native Spanish speakers, conducted the analysis in Excel, and data were analysed in the original language of the survey responses. Firstly, the first two authors independently conducted inductive coding of all anonymised responses and generated their own preliminary codebooks. Next, they compared and discussed both sets of emerging codes and their broader thematic categorisations, reconciling their two initial codebooks in order to develop a single harmonised codebook, which contained 24 codes for the sample that reported change towards greater endorsement of legal abortion and 16 codes for the sample that reported change towards lower endorsement of legal abortion. During this reconciliation phase, efforts were made to reduce the number of codes for the final codebook. Thirdly, both authors independently coded all the open-ended responses again using the final codebook. Fourthly, inter-rater reliability was calculated using Cohen’s kappa. Results reflected a high degree of agreement (see the codebook and inter-rater reliability results in Table A3 in the Appendix). Fifthly, the remaining rare disagreements between coders were resolved on a case-by-case basis through discussion. Sixth, the first two authors then conducted content analysis based on the identified themes that emerged from the inductive coding.

#### Multivariate analysis

Lastly, we used multinomial logistic regression models to identify relevant predictors of people’s change in abortion attitudes before and after the *Dobbs v. Jackson* decision. The two dependent variables were derived from participants’ responses to our measures of attitudinal change as described earlier – that is, (1) the perceived measure of change; and (2) the measure of change across waves. As a result, for the measure of change across waves we identified change towards (1) greater endorsement of legal abortion; (2) no change in attitudes towards legal abortion; or (3) lower endorsement of legal abortion.

In the multinomial logistic regression models the no change group was used as the reference category. Thus, we assessed (1) what predicted greater endorsement of legal abortion compared with those who endorsed no change, and (2) what predicted lower endorsement of legal abortion compared with those who endorsed no change. In addition to abortion identity labels, the independent variables included in the analyses were the sociodemographic characteristics aforementioned.

All analyses, including data cleaning, were completed in Stata 16. To ease interpretation, we used the margins command in Stata to examine the average marginal effects (AMEs). Results from the models are presented as AMEs, which represent the average effect of a change in an independent variable on the dependent variable.^29,30^ In our models, the AMEs represent the change in the probability of participants reporting greater or lower endorsement of legal abortion after *Dobbs*, compared with no change, for a change in the independent variable. Results containing the relative risk ratios (RRRs) are also included in Table A4 in the Appendix.

### Research team composition and reflexivity

The research team consists of six researchers: four cis-gender women, and two cis-gender men. KJ, RT, and BC self-identify as White non-Hispanic, LMM and XB as Latinas, and WJL as Asian. The former three are foreign-born and immigrated from El Salvador, Spain, and Taiwan, respectively. All six authors reside in the United States (KJ, BC, and LMM in Indiana, RT and WJL in Arkansas, and XB in Massachusetts). The team has an interdisciplinary composition that includes backgrounds in sociology (BC, LMM, and XB), demography (LMM and XB), education (RT and WJL), psychology (RT and WJL), behavioural science (KJ), and criminology (BC). All six of us collaborated on a large project focused on developing survey measures of abortion attitudes in the US from a value-neutral perspective. To that end, our project is advised by multiple groups, including movement leaders from both the pro-choice/reproductive justice movement and pro-life/anti-abortion movement. While we strive to maintain neutrality in our research and data collection efforts, we recognise that our personal perspectives on abortion may unintentionally influence our interpretation of the data and discussion of the findings. To mitigate potential individual biases, we held regular team discussions to critically examine emerging interpretations and challenge underlying assumptions. This reflexive process supported our efforts to maintain analytical objectivity and ensured that our findings remained grounded in the participants’ narratives.

## Results

### Awareness and agreement/disagreement with the decision to overturn *Roe v. Wade*

**Figure 1.**
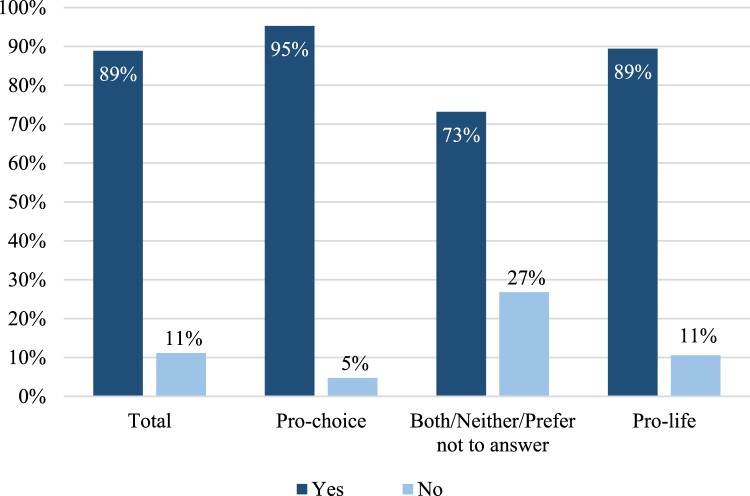
Awareness of the *Dobbs v. Jackson* decision. *Before taking this survey, had you heard that *Roe v. Wade* was overturned (gotten rid of) in June 2022?* (*n* = 719)

Overall, weighted results from Figure 1 indicate that almost 90% of the 734 respondents were aware that *Roe v. Wade* was overturned in June 2022. However, those who identified as pro-choice (*n* = 358) were the most aware (95%) compared with pro-life participants (*n* = 208, 89% of awareness) and especially compared with those who identified as both/neither/prefer not to answer (*n* = 153, 73% of awareness) (*χ*^2^ (2) = 52.79, *p* < .001).

**Figure 2.**
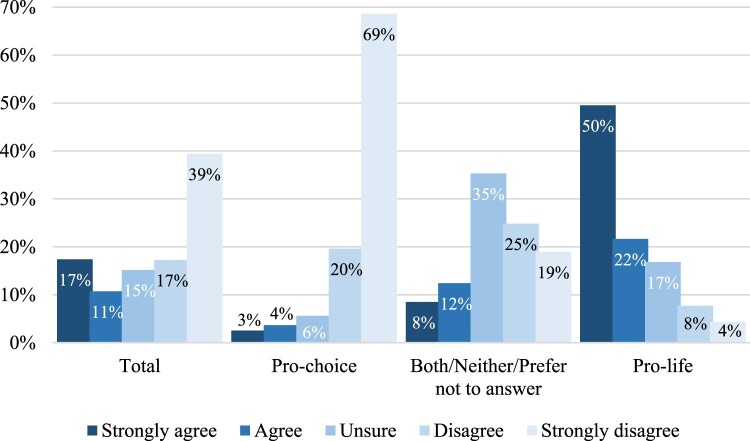
Agreement with the *Dobbs v. Jackson* decision. *Do you agree or disagree with the decision to overturn (get rid of)* Roe v. Wade? (*n* = 718)

As Figure 2 shows, we found that overall, the majority of respondents (*n* = 441, 56%) disagreed or strongly disagreed with the decision, with some differences by abortion identity worth noting (*χ*^2^(8) = 454.30, *p* < .001). Specifically, 89% of respondents identifying as pro-choice said they disagreed or strongly disagreed with the decision to overturn *Roe v. Wade* (*n* = 315), and by contrast, the percentage of people identifying as pro-life who agree or strongly agree was comparably lower (*n* = 148, 72%), but still the majority. The group that identified as both/neither/prefer not to answer is by far the most complex in their sentiment towards the decision, however, more people in this group tended to disagree (*n* = 67, 44%) than agree (*n* = 32, 20%) with the overturn of *Roe v. Wade*.

### Changes in attitudes towards abortion after *Dobbs v. Jackson*

We measured the impact of the *Dobbs v. Jackson* decision on people’s abortion attitudes through two different approaches: (1) a perceived measure of change, by directly asking participants in Wave 2 if their views towards the legality of abortion changed as a result of the *Dobbs* decision, and (2) a measure of response change across waves, by comparing responses to the same item about abortion legality in Wave 1 and Wave 2.

**Figure 3.**
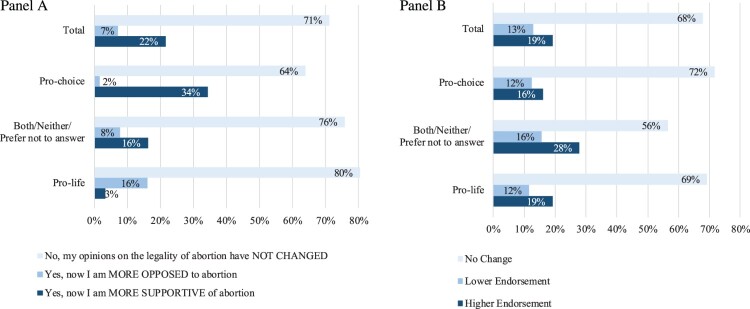
Participants’ change in attitudes towards abortion legality by identification with abortion identity labels. *Panel A: Perceived change (Wave 2), Have your opinions about the legality of abortion changed after *Roe v. Wade* was overturned (gotten rid of)? (n* *=* *716) Panel B: Response change across waves (Wave 1 and 2), Do you think abortion should be legal in all cases, legal in most cases, illegal in most cases, or illegal in all cases? (n* *=* *694)*

Firstly, as Figure 3 (Panel A) shows, overall, 22% of respondents (*n* = 166) self-reported that their views changed towards greater endorsement of legal abortion compared with before the decision. Notably, the proportion of participants who changed towards greater endorsement is three times higher than the proportion stating that they changed towards lower endorsement after the *Dobbs* decision (*n* = 57, 7%). In this case, we also identified differences across abortion identity labels (*χ*^2^(4) = 103.96, *p* < .001). We also observed that the decision strengthened the stance of those who identified as pro-choice more than it did for those who identified as pro-life. In other words, while 34% of pro-choice respondents reportedly changed towards greater endorsement of legal abortion after *Dobbs* (*n* = 123), only 16% of pro-life respondents changed towards lower endorsement after *Dobbs* (*n* = 33). Similarly, to the results from Figure 2, those who identified as both/neither/prefer not to answer tended to report having changed more towards greater endorsement (*n* = 25, 16%) than towards lower endorsement (*n* = 12, 8%).

Secondly, as Figure 3 (Panel B) shows, overall, 19% of participants changed towards greater endorsement of legal abortion (*n* = 134) and 13% changed towards lower endorsement of legal abortion after the decision (*n* = 89). These results align with the trend observed for the measure of perceived change aforementioned, although the difference between the greater endorsement and lower endorsement groups is smaller for the measure of change across waves than perceived change. Figure 3 (Panel B) also shows smaller differences across abortion identity labels (χ2(4) = 11.99, *p* = .017). Notably, the measure of response change across waves (Figure 3 (Panel B)) shows that all three groups – pro-choice (*n* = 57, 16%), both/neither/prefer not to answer (*n* = 39, 28%), and pro-life (*n* = 38, 19%) – indicated greater endorsement towards legal abortion after the *Dobbs* decision when comparing participants’ responses in Wave 2 to their responses from Wave 1 (e.g. changing from illegal in most cases to legal in most cases). In contrast, only 12% (*n* = 44), 16% (*n* = 22), and 12% (*n* = 23) of pro-choice, both/neither/prefer not to answer, and pro-life participants respectively reported that they changed towards lower endorsement of legal abortion in Wave 2 compared with Wave 1 (e.g. illegal in most cases to illegal in all cases). Stated differently, disregarding abortion identity, more respondents after than before *Dobbs* thought abortion should be legal in more circumstances compared with respondents who thought the opposite – that there should be more circumstances in which abortion should not be legal. A more detailed representation of the data included in Figure 3 (Panel B) can be found in Figure A1 in the Appendix using alluvial plots.

### Reasons behind self-reported changes in people’s abortion attitudes after *Dobbs v. Jackson*

Respondents who self-reported greater endorsement (*n* = 158) and lower endorsement (*n* = 53), were asked to explain, in their own words, why their views on legal abortion changed after the *Dobbs* decision. Among participants, 15.2% of those who reported greater endorsement and 26.4% of those who reported lower endorsement left the question blank, which left us with 134 and 39 valid open-ended responses, respectively.

Table 1 presents the main themes generated from the inductive analysis of open-ended responses by abortion identity subgroups. Most respondents who reported change towards greater endorsement and provided a content response identified as pro-choice (68.4%), and the plurality of those who reported change towards lower endorsement identified as pro-life (45.3%). A few pro-life respondents (*n* = 7; 4.4%) reported change towards greater endorsement, and a few pro-choice respondents (*n* = 4; 7.5%) reported change towards lower endorsement.

**Table 1.** Presence of themes in survey open-ended responses by abortion identity labels

Greater Endorsement						
	*N* (%)	A woman’s right	Consequences of *D v. J* Decision	Against government involvement	Personal views on abortion	IDK/Invalid
**Total**	158 (100%)					
Total with content response	134 (85%)	57%	31%	25%	20%	2%
Pro-choice	108 (68%)	59%	33%	24%	18%	3%
Both/Neither/Prefer not to answer	19 (12%)	42%	16%	37%	26%	0%
Pro-life	7 (4%)	57%	29%	14%	43%	0%
Blank	24 (15%)	–	–	–	–	–
**Lower Endorsement**	** **					
	**N (%)**	**Moral views against abortion**	**Legal views about abortion**	**Judgement on women**	**IDK/Invalid**	
**Total**	53 (100%)					
Total with content response	39 (73.6%)	62%	21%	15%	8%	
Pro-choice	4 (7.5%)	0%	50%	0%	50%	
Both/Neither/Prefer not to answer	11 (20.8%)	46%	18%	27%	9%	
Pro-life	24 (45.3%)	79%	17%	13%	0%	
Blank	14 (26.4%)	–	–	–	–	–

Among respondents who changed towards greater endorsement, we identified four main themes: (1) A woman’s right, (2) Consequences of *Dobbs v. Jackson*, (3) Against government involvement, and (4) Personal views on abortion. Firstly, more than half (56.7%) of respondents who expressed having changed towards greater endorsement of legal abortion argued primarily that the *Dobbs* decision strengthened their belief that abortion is a personal decision or that overturning *Roe v. Wade* was a step backward for women’s rights, a sentiment shared by more than half of those who identified as pro-choice (59.3%) and pro-life (57.1%). For example, a 24-year-old, White non-Latinx, pro-choice female, said, “It’s like going backwards. We made progress then took it away”. Secondly, almost a third of respondents (30.6%) expressed concerns regarding the potential consequences of the *Dobbs* decision and how the decision made them realise the importance of abortion. For example, some of these concerns were related to the future safety of abortion, how other fundamental rights might be at risk, how the decision may exacerbate inequalities among women across the country and may worsen the situation for the most vulnerable communities, or outrage generated by some states passing abortion bans throughout pregnancy (with limited exceptions). A 26-year-old, White non-Latinx female, who identified as pro-life shared, “I didn’t expect to get a strong feeling about it but even as someone who is pro-life, I realized that I felt it was wrong to take away access to safe, legal abortions, especially if there are no exceptions”. Notably, the concerns listed above related to the future consequences of the *Dobbs* decision were more present among respondents who identified as pro-choice (33%), and also pro-life (29%), but less common among those who identified as both/neither/prefer not to answer (16%). Thirdly, slightly more than a quarter of participants who changed towards greater endorsement (25%) explicitly positioned themselves against government involvement in women’s lives, expressed their distrust of the government, and indicated outrage over the politicisation of the Supreme Court. Of note, this theme was more prevalent among respondents who identified as both/neither/prefer not to answer (37%) compared with pro-choice (24%) or pro-life respondents (14%). For example, a 36-year-old, multiracial female, who preferred not to answer the abortion identity item shared, “Before, the choice was given to the ppl [people], now it’s given to the state, which is not for the ppl [people] but interest groups”. Fourthly, 20% of respondents disagreed with the decision because they believed it goes against their personal views on abortion, both in terms of abortion circumstances (e.g. there are infinite reasons for having an abortion) or gestational timing (e.g. restrictive laws will generate human suffering). For example, a 28-year-old, White non-Latinx, pro-choice male, expressed, “Before I was more open to late term restrictions. But the more I learn about late term abortions and medical situations that occur I no longer support any restrictions. Abortion should be an option at all stages of pregnancy to reduce human suffering”.

Among respondents who changed towards lower endorsement of legal abortion, we identified three main themes: (1) Moral views against abortion, (2) Legal views about abortion, and (3) Judgement on women. Firstly, respondents most commonly expressed that moral views against abortion were now legitimised by the *Dobbs* decision (62%), such as the belief that abortion means taking a life, that life starts at conception, or that the rights of the unborn need to be protected. For instance, a 78-year-old, White non-Latinx, pro-life female, said, “I believe it is wrong to kill a baby even if it is only 1 d old or full term”. Of note, 79% of pro-life respondents expressed this kind of sentiment, and 46% of those who identified as both/neither/prefer not to answer, but none of the four pro-choice respondents who changed towards lower endorsement provided reasonings that were related to moral views. Secondly, approximately a fifth (21%) of respondents mentioned having changed towards lower endorsement due to legal reasons, such as their preference for states to regulate abortion or their desire to see more restrictions in place. Two of the four pro-choice respondents expressed this sentiment. As simply stated by a 40-year-old, Black, pro-choice female, “[Abortion] needs more regulation”. Thirdly, 15% of participants who changed towards lower endorsement provided explanations that pertained to the judgment of women’s behaviours. For example, because they want women to “be more responsible of their acts” or because they believe some women use abortion “for convenience” or “as a birth control measure”. As an example, a 61-year-old, Latinx female, who identified as both pro-life and pro-choice wrote, “[porque las] mujeres adultas deben tener responsabilidad de sus hechos … por el hecho de que ya no les gustó quien participa en su embarazo deciden abortar fácilmente.”^†^

### Abortion identity as a predictor of attitudinal change after the *Dobbs v. Jackson* decision

Lastly, we explored whether self-identification with abortion identity labels explains changes in people’s abortion attitudes in response to the *Dobbs* decision. We used multinomial regression models to predict changes towards greater or lower endorsement of legal abortion compared with reporting no change in abortion attitudes. We computed two sets of models, one set using the perceived measure of change (*r*^2^ = .17, *χ*^2^(36) = 165.31, *p* < .001) presented in Figure 3 (Panel A) (representing Models 1 and 3 in Table A4) and a second set using the measure of response change across waves (*r*^2^ = .07, *χ*^2^(36) = 75.18, *p* < .001) presented in Figure 3 (Panel B) (representing Models 2 and 4 in Table A4). We controlled for a set of sociodemographic characteristics (gender, age, race/ethnicity, education, church attendance, party identification, urbanicity, and US region of residency).

**Figure 4.**
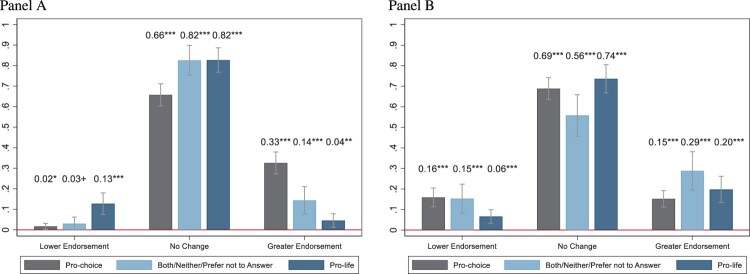
Probability of change in abortion attitudes after *Dobbs v. Jackson* based on abortion identity. *Panel A: Perceived measure of change Panel B: Response change across waves*

Figure 4 represents the predicted probabilities of attitudinal change after the *Dobbs* decision by abortion identity groups. Most respondents did not change their attitudes after the *Dobbs* decision. Figure 4 (Panel A) represents attitudinal change based on the perceived measure of greater/lower endorsement of legal abortion. Results indicate pro-choice participants perceived attitudinal change in the direction of greater endorsement for legal abortion (pr = 0.33, *p* < .001). As seen in the descriptive results, the both/neither/prefer not to answer group perceived more change in attitudes towards greater endorsement (pr = 0.14, *p* < .001) rather than lower endorsement (pr = 0.03, *p* = .061) of legal abortion. Notably, we observe greater variability in the extent that subgroups experienced a change in attitudes when accounting for the response change across waves variable (Figure 4: Panel B). In this instance, the both/neither/prefer not to answer and pro-choice sub-groups were more likely to experience attitudinal change, predominantly towards greater endorsement (pr = 0.29, *p* < .001; pr = 0.15, *p* < .001, respectively), but also slightly towards lower endorsement (pr = 0.15, *p* < .001; pr = 0.16, *p* < .001, respectively). Finally, results indicate that those within the pro-life group who changed their views towards legal abortion tended to change towards greater endorsement (pr = 0.20, *p* < .001).

**Figure 5.**
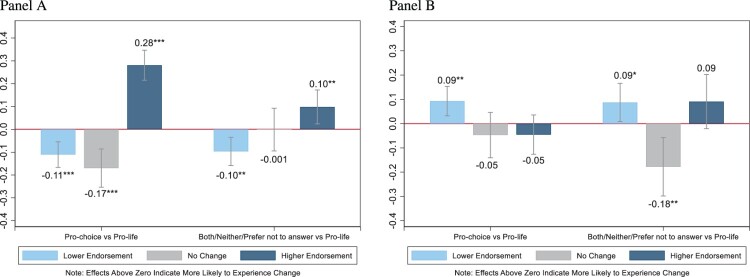
Average marginal effects of abortion identity on change in abortion attitudes after *Dobbs v. Jackson.Panel A: Perceived measure of changePanel B: Response change across waves*

Average marginal effects are also represented in Figure 5 and indicate that response patterns differed based on our measure of change. When respondents were asked broadly regarding their endorsement of legal abortion (perceived measure of change, Panel A), those who identified as pro-choice (Δ** = −0.11, 95% CI: −0.17 to −0.05, *p* < .001), and both/neither/prefer not to answer (Δ = −0.10, 95% CI: −0.16 to −0.03, *p* = .002), were less likely to report having changed towards lower endorsement of legal abortion after *Dobbs* compared with pro-life respondents. As expected, those who identified as pro-choice (Δ = 0.28, 95% CI: 0.21–0.35, *p* < .001), and to a lesser extent, both/neither/prefer not to answer (Δ = 0.10, 95% CI: 0.02–0.17, *p* = .010), were more likely to report having changed towards more endorsement of legal abortion after *Dobbs* compared with pro-life respondents. Interestingly, when we compared their responses across waves (measure on response change across waves, Panel B), we found that both the pro-choice (Δ = 0.09, 95% CI: 0.03–0.15, *p* = .003) and both/neither/prefer not to answer (Δ = 0.09, 95% CI: 0.01–0.17, *p* = .030) groups were more likely to have changed towards lower endorsement after *Dobbs* compared with pro-life respondents.

### Other predictors of change on abortion attitudes after the *Dobbs* decision

Beyond examining the predictive power of abortion identity labels on attitudinal change, our analysis extends to exploring how various control variables influenced changes in abortion attitudes. AMEs derived from our models are presented in Tables 2 and 3, corresponding to the perceived measure of change and the measure of change across waves, respectively.

**Table 2.** Average marginal effects from multinomial regression models for *perceived measure of change* in abortion attitudes after *Dobbs v. Jackson* (*n* = 642)

	Lower Endorsement (*n* = 35)			No Change (*n* = 465)			Greater Endorsement (*n* = 142)		
	AME		SE	AME		SE	AME		SE
**Abortion identity (Ref. Pro-life)**									
Pro-choice	−0.11	***	0.03	−0.17	***	0.04	0.28	***	0.03
Both/Neither/Prefer not to answer	−0.10	**	0.03	−0.001		0.05	0.10	**	0.04
**Gender (Ref. Man)**									
Woman	−0.01		0.02	0.06	+	0.03	−0.05		0.03
**Age**	0.00		0.00	0.00		0.00	0.00		0.00
**Race/ethnicity (Ref. White)**									
Latinx	0.14	***	0.04	−0.19	***	0.06	0.05		0.05
Black/African American	0.05		0.05	−0.06		0.07	0.01		0.05
Multiracial/Other	0.04		0.04	−0.06		0.07	0.02		0.06
**Education (Ref. High School or less)**									
Some college	0.00		0.02	0.04		0.04	−0.04		0.04
Bachelor or higher	0.00		0.02	0.03		0.04	−0.03		0.04
**Church attendance (Ref. Weekly/Monthly)**									
Yearly	−0.01		0.02	−0.05		0.05	0.06		0.04
Never	−0.02		0.02	−0.04		0.05	0.06		0.04
**Party identification (Ref. Republican)**									
Democrat	−0.04		0.02	−0.01		0.05	0.05		0.05
Other/Any	−0.01		0.02	−0.01		0.05	0.02		0.04
**Urbanicity (Ref. Rural)**									
Urban	−0.01		0.03	−0.02		0.05	0.03		0.04
Suburban	−0.01		0.03	−0.09	+	0.05	0.10	**	0.04
**Region (Ref. South)**									
Northeast	0.04		0.03	0.02		0.05	−0.06		0.04
Midwest	0.06	*	0.03	−0.04		0.05	−0.02		0.04
West	0.03		0.02	0.07		0.05	−0.10	**	0.04

**Table 3.** Average marginal effects from multinomial regression models for *measure of response change across waves* in abortion attitudes after *Dobbs v. Jackson* (*n* = 626)

	Lower Endorsement (*n* = 77)			No Change (*n* = 431)			Greater Endorsement (*n* = 118)		
	AME		SE	AME		SE	AME		SE
**Abortion identity (Ref. Pro-life)**									
Pro-choice	0.09	**	0.03	−0.05		0.05	−0.05		0.04
Both/Neither/Prefer not to answer	0.09	*	0.04	−0.178	**	0.06	0.09		0.06
**Gender (Ref. Man)**									
Woman	−0.01		0.03	−0.04		0.04	0.05	+	0.03
**Age**	0.00		0.00	0.00		0.00	0.00		0.00
**Race/ethnicity (Ref. White)**									
Latinx	0.03		0.04	−0.10		0.06	0.07		0.05
Black/African American	0.04		0.05	−0.10		0.07	0.06		0.06
Multiracial/Other	−0.03		0.04	0.09		0.06	−0.06		0.05
**Education (Ref. High School or less)**									
Some college	−0.04		0.04	0.06		0.05	−0.01		0.04
Bachelor or higher	−0.06	+	0.03	0.09	+	0.05	−0.03		0.04
**Church attendance (Ref. Weekly/Monthly)**									
Yearly	−0.05		0.04	0.06		0.05	−0.01		0.04
Never	−0.11	**	0.04	0.09	+	0.05	0.02		0.04
**Party identification (Ref. Republican)**									
Democrat	−0.06		0.04	0.10	+	0.05	−0.04		0.05
Other/Any	−0.02		0.04	0.02		0.05	0.00		0.04
**Urbanicity (Ref. Rural)**									
Urban	0.01		0.04	−0.06		0.06	0.05		0.05
Suburban	−0.02		0.04	0.01		0.05	0.01		0.04
**Region (Ref. South)**									
Northeast	−0.07	*	0.03	0.09	+	0.05	−0.02		0.04
Midwest	−0.01		0.04	−0.04		0.05	0.05		0.04
West	0.04		0.04	−0.04		0.05	0.01		0.04

Relative to abortion identity labels, other variables exhibited more modest predictive capacities. According to the perceived measure of change (Table 2), respondents who identified as Latinx, compared with White, had a 0.14 higher probability (95% CI: 0.06–0.22, *p* < .001) of having changed towards lower endorsement of legal abortion, and those residing in the Midwest, compared with the South, had a 0.06 higher probability (95% CI: 0.01–0.12, *p* = .022) of having changed towards lower endorsement. Conversely, for attitudinal changes across survey waves (Table 3), people who never attended church services, relative to regular churchgoers, had a 0.11 lower probability (95% CI: 0.03–0.19, *p* = .005) of having changed towards lower endorsement of legal abortion. Similarly, residents of the Northeast had a 0.07 lower probability (95% CI: 0.00–0.13, *p* = .037) of changing towards lower endorsement than their Southern counterparts.

We also identified predictors in the models assessing the perceived measure of change. Notably, participants who resided in suburban locations, compared with rural ones, had a 0.10 greater probability (95% CI: 0.02–0.18, *p* = .011) of having changed towards greater endorsement of legal abortion. Conversely, people living in the West, compared with the South, had a 0.10 lower probability (95% CI: 0.02–0.18, *p* = .013) of having changed towards greater endorsement. Additionally, not experiencing a change in abortion attitudes was associated with ethnic and geographic factors. Specifically, people who identified as Latinx had a 0.19 lower probability (95% CI: 0.08–0.31, *p* < .001) of maintaining their pre-*Dobbs* attitudes towards abortion compared with White respondents.

## Discussion

### Awareness of and agreement with the *Dobbs* decision

In the months following *Dobbs v. Jackson*, according to our bivariate analysis, our sample was highly informed of the decision. Nine in ten participants were aware *Roe v. Wade* was overturned. This was especially true for participants who identified as pro-life or pro-choice whereas those identifying as both/neither/prefer not to answer were less aware. In contrast to the rather low awareness of the case immediately before and after the *Dobbs* decision,^31^ it is notable that such a high proportion of participants were aware of the decision in the months following the case. This discrepancy could be attributed to significant media attention regarding the decision,^32–34^ subsequent changes in state legislation in the months following,^35^ and an increase in interest in abortion and reproductive health potentially in response to the decision.^36,37^

We also observed high levels of uncertainty regarding agreement with *Dobbs*. Those who identified as both/neither/prefer not to answer expressed the highest levels of uncertainty, followed by pro-life respondents and a small number of pro-choice respondents. Higher levels of uncertainty among pro-life and both/neither/prefer not to answer respondents may denote complexity in people’s attitudes and beliefs regarding abortion and their identification with these labels.^3,22^ People may hold certain beliefs regarding abortion, encouraging their identification with certain labels that may conflict with other aspects of their beliefs – such as a pro-choice identifying person endorsing abortion restrictions and a pro-life identifying person expressing disapproval or uncertainty regarding the *Dobbs* decision.

### Change in abortion attitudes after the *Dobbs* decision

Our findings reveal that most people’s opinions on abortion remained unchanged post-*Dobbs*. This observation is consistent with established theoretical frameworks suggesting that once people form an opinion on a sensitive issue like abortion, their attitudes are hard to change.^11^ Additionally, and specifically related to Supreme Court rulings, our findings also align with research that asserts that when people accept a decision such as the landmark ruling in *Roe v. Wade*, subsequent decisions, such as *Dobbs v. Jackson*, yield minimal change.^16^

Notably, though, among those who did experience a change in attitudes, we found that people reported greater endorsement of legal abortion after the *Dobbs* decision. This result aligns with post-*Dobbs* poll findings from Gallup.^38^ By examining open-ended responses, we were able to delve deeper into participants’ perspectives. We highlight two contrasting patterns of why people’s attitudes changed towards greater endorsement of legal abortion. Firstly, some participants described the ruling as an erosion of personal rights, government intrusion into individual autonomy, and the potential ramifications for abortion safety, healthcare access, and broader health outcomes. These findings are supported by the theories of attitudinal change, particularly the aforementioned active updating model that explains that people might change their attitudes as they are exposed to new information, knowledge, and events.^9,12^ Regarding the influence of Supreme Court decisions on public opinion, theories suggest that the Court’s perceived credibility can significantly intensify public opinion following a contentious ruling, prompting people to solidify their stance either in support of or in opposition to the decision.^15^ We contend that the resurgence of vivid discourse on abortion in mass media following the *Dobbs* decision,^39^ coupled with the subsequent dissemination of information to the public, (e.g.^33,40^) may have led to a sudden surge in people reacting to the decision,^41^ raising awareness of the outcomes of banning or restricting abortion,^42^ and to people re-examining their beliefs about the issue.^43^

Conversely, participants who reported changing towards lower endorsement of legal abortion highlighted how the Court’s decision reinforced their prior views, particularly regarding their belief in defending the rights of the unborn. This perception may align with traditional theories on the effect of Supreme Court decisions in the US, such as the positive response hypothesis.^13^ According to this hypothesis, public opinion tends to align with and accept a Court decision, viewing judges as the ultimate decision-makers in a controversial debate. However, some of our participants who changed towards lower endorsement of legal abortion, including some who identified as pro-choice, expressed the desire for additional abortion regulation, even prior to *Roe v. Wade* being overturned. This sentiment, previously identified in the literature on abortion attitudes,^44,45^ reflects a nuanced stance: while participants may not advocate for outright illegality, they express a desire for stricter regulations that could potentially limit access.

Our results also indicated differences across abortion identity labels. We found that people who identified as both/neither/prefer not to answer distinctly leaned towards greater disagreement with the *Dobbs* decision and changed towards greater rather than lower endorsement of legal abortion. This is important because it speaks to the limitations of categorising people dichotomously by abortion identity labels (i.e. pro-life v. pro-choice). It may also speak to the potential effect the decision had on sub-groups of people whose opinions are more malleable (i.e. people who identified as both/neither/prefer not to answer). Perhaps the decision prompted a change among those who are more uncertain or ambivalent towards legal abortion. Further, when assessing changes across waves, some pro-life participants changed their views towards greater endorsement of legal abortion and some pro-choice respondents changed towards lower endorsement of legal abortion. Such findings underscore previous research, (e.g.^3,5,22^) signalling complexity in beliefs held regarding abortion across different frameworks (e.g. morality vs. legality vs. labels). These surprising shifts seemed slightly more prevalent among pro-life participants compared with the other sub-groups, which could be conceived as a “loss” for the pro-life movement. Consistent with Ura’s^2^ articulation of the thermostatic model of the public mood, perhaps the Supreme Court’s decision inspired some to react in opposition to their previously held position on abortion or highlighted the multi-dimensional ways that people consider abortion^31^.

### Nuances in measuring attitudinal change in surveys

In exploring change in abortion attitudes, we evaluated two different measures: (1) respondents’ self-reported perception of change in abortion attitudes after the *Dobbs* decision and (2) a comparison of the same survey item across the two waves before and after the decision. The total sample shows higher levels of change towards greater endorsement of legal abortion for both measures. Previous studies on perceived change in abortion attitudes indicate that exposure to knowledge and information can trigger attitudinal change^46^. It is possible that greater exposure to media and news on abortion during the months following the decision contributed to respondents’ greater engagement with information from different sources, resulting, for example, in greater endorsement of legal abortion^47–50^.

In the case of the perceived measure of change, the percentage of participants who identified as pro-choice and both/neither/prefer not to answer and changed towards greater endorsement was three times higher than lower endorsement, but this pattern did not persist for those who identified as pro-life. Yet, we want to highlight two other novel observations. Firstly, the *Dobbs* decision had a stronger effect on pro-choice than both/neither/prefer not to answer identifying participants. This might not be surprising given that overturning *Roe v. Wade* was perceived as a direct attack on the pro-choice movement’s ideals. In contrast, the decision might not have had the same effect on participants who felt ambivalent about abortion or those who did not have a strong stance on the issue beforehand. Secondly, and similarly, when looking at the results using the measure of response change across waves, the percentage of participants who changed towards greater endorsement was nearly one and a half times higher compared with those who changed towards lower endorsement. More specifically, the *Dobbs* decision had a stronger effect on both/neither/prefer not to answer and pro-life identifying participants. This may be because the post-*Dobbs* changes led to substantial reductions in abortion access, further reinforcing the strong beliefs already held by those who identify as pro-choice. Alternatively, the change in public policy may have instigated those holding an anti-abortion perspective towards more change because the change in the status quo could have challenged the attitudes and beliefs they held.

However, when comparing across measures, it is interesting that the measure using response change across waves shows that 12% of pro-choice respondents changed towards lower endorsement of legal abortion after the decision, compared with only 2% in the perceived measure of change. Likewise, 19% of pro-life respondents reported greater endorsement towards legal abortion after *Dobbs*, compared with only 3% when using the measure of perceived change. In other words, the measure of response changes across waves reports greater variability of attitudinal change than the perceived measure of change, as shown in Figure 3. Several factors might explain these descriptive differences. Firstly, it is possible that we were unable to capture more nuanced attitudes because the general measure of abortion legality in our perceived measure of change assessment did not account for gestational timing and/or abortion circumstance. Secondly, the discrepancy in change across the two measures can also be explained methodologically by the measures’ characteristics: the more detailed response options (i.e. four) in the response change across waves measure allowed for greater variability (e.g. legal/illegal in all/most cases) compared with the binary response options of the perceived measure of change (i.e. “Yes, now I am more supportive of abortion being legal” vs. “Yes, now I am more opposed to abortion being legal”). The greater variability in the response change across wave could have therefore been more sensitive to change. Thirdly, some people may have interpreted the two measures differently even though both specifically asked about the legality of abortion in the question stem. Notably, the response options for the perceived measure of change mentioned legality at the end (i.e. “Yes, now I am more supportive of/opposed to abortion being legal”), while the response options in the measure of response change across waves mentioned legality at the beginning (i.e. “legal/illegal in most/all cases”). As a result, it could be that the perceived self-reported measure might have been interpreted more vaguely by low-effort (or lower-effort) respondents who might have paid attention to only the beginning of the answer, thus interpreting it more broadly (e.g. abortion as a moral rather than a legal issue) than the measure of response change across waves. Highlighting this difference is important because it might denote how people’s attitudes towards abortion might emerge from different frameworks (e.g. morality, legality) even when they are being asked about similar constructs (e.g. abortion legality).^5^ Finally, these results may suggest that respondents’ reported survey perceptions might not necessarily align with responses to less subjective survey items, or it may be the case that some people feel so conflicted regarding abortion that they might answer one or the other way on two similar, yet different questions. For example, our analysis of open-ended responses suggests that some people reporting lower endorsement of legal abortion after *Dobbs* would like to see more restrictions on abortion, given their moral views on the topic, but do not necessarily want to make abortion illegal. Such findings may once again, underscore the complexity embedded in abortion attitudes.

Despite this, the multinomial regression models for response change across waves identified fewer associations compared with the models for the perceived measure of change. In light of this, it is possible that response changes across waves were more random or uniformly distributed across groups, whereas perceived changes tended to be more concentrated among certain groups (e.g. racial/ethnic groups, urbanicity, regional groups). The discrepancy in their predictive capacity highlights the importance of including multiple measures to assess attitudinal change in survey research.

### Limitations

Firstly, as noted earlier, some observed patterns in this study might be the result of different ways our respondents conceptualised our survey questions. Employing cognitive interviewing, which examines how respondents interpret certain survey items, would therefore be fruitful as it helps identify potential misunderstandings, ambiguities, or biases in question wording. Secondly, the multilingual component of our survey is a strength but entails a limitation in this particular analysis. Spanish-speaking adults in the US are not as familiar with the terms pro-life/pro-choice and their corresponding translations (pro-vida/pro-elección)^25^ and demonstrate more variability in their conceptualisation of these terms compared with English-speakers.^24^ Thus, there may be some misalignment in abortion attitudes and abortion attitude labels among some of the Spanish-speaking respondents. Finally, due to a strong majority of our sample exhibiting either no change in endorsement or greater endorsement of legal abortion after the *Dobbs v. Jackson* decision, our sample sizes for lower endorsement for both the perceived measure of change and the response change across waves were small. We want to retain these groups in the analysis as their comparative perspective is an important component to understanding differences due to the type of change. However, such results should be interpreted with caution.

### Health and policy implications

These findings have important policy as well as measurement related implications. Notably, people, especially larger proportions of those with ambivalent or mixed views (i.e. both/neither/prefer not to say), were more likely to disagree with the *Dobbs* decision. And although we did not see change in attitudes among the majority of the sample, among those that did change, there was greater endorsement of legal abortion. Because *Dobbs* returned decisions regarding the legal status of abortion back to states, state-level lawmakers may consider such sentiment. As we have already seen, it may be the case that individual states contend with this via state-level ballot initiatives.

From a policy perspective, a better understanding of public sentiment regarding the *Dobbs* decision and potential changes in attitudes towards abortion can help inform lawmakers. This is especially relevant at the state level, where numerous abortion-related ballot initiatives have emerged since *Dobbs*.

Finally, such findings underscore the importance of precise measurement in assessing abortion attitudes and perhaps attitudes towards other social issues more generally. The discrepancies that emerged in measures of perceived versus measured change are important to highlight as a reminder to researchers to carefully select their measures of interest to address the aims of their study.

## Conclusion

We examined the influence of the *Dobbs v. Jackson* decision on peoples’ attitudes towards legal abortion, considering people’s identification with abortion labels. While our findings corroborated the expected alignment between people’s identities and their respective attitudes towards abortion generally, they also uncovered significant insights for people whose views do not strictly adhere to the conventional meanings represented by these labels in the US abortion context. Specifically, for those who identified with the categories both/neither/prefer not to answer, we observed a change towards greater endorsement of legal abortion post-*Dobbs*. Results from this study highlight the critical need for a nuanced approach in measuring abortion attitudes, moving beyond simplistic binary frameworks to better capture the diverse spectrum of opinions that exist within the complex terrain of the abortion debate.

Overturning *Roe v. Wade* fundamentally altered the legal landscape of abortion in the US. The accuracy of public opinion surveys in reflecting the true sentiments of the general population towards abortion is crucial for the future of abortion access in the country. This is significant for three main reasons: firstly, the potential for public opinion surveys to shape the broader societal views on abortion; secondly, the likelihood that policymakers and state legislatures will rely on these public sentiments to guide future abortion regulations at the state level; and thirdly, the possibility of public opinion influencing and shaping previous and upcoming state constitutional amendments regarding abortion through ballot initiatives. As such, it is essential for researchers, media, and polling organisations to consider the complexity of people’s attitudes towards abortion when conducting studies and reporting on findings.

## Supplementary Material

Appendix

## Footnotes

* The statement read as follows: “*Roe v. Wade* was the 1973 Supreme Court decision stating that a woman had a constitutional right to choose to have an abortion until she was about 24 weeks pregnant. *Roe v. Wade* was overturned (gotten rid of) in June 2022. The following questions will ask about *Roe v. Wade* and current abortion laws. Please answer them to the best of your ability.”

† “because adult women should take responsibility for their acts … because they no longer like who participates in their pregnancy they decide to get an abortion easily.”

** Δ = This represents the difference in average predicted probability between the reference group (*pro-life*) and comparison groups (e.g., *pro-choice*, *both/neither/prefer not to answer*).

## Provenance statement

This article was not commissioned and went through external peer review.
