# Supplementary material for: Assessing the impact of the Dobbs v. Jackson decision on abortion attitudes by abortion identity labels: a mixed-methods longitudinal study
Source: Sex Reprod Health Matters. 2025 Jun 16;33(1):2518669. doi: 10.1080/26410397.2025.2518669 (PMC12302401; doi:10.1080/26410397.2025.2518669)

**Table A1.** Sample characteristics

|  |  | **Pro-choice** | | **Both/Neither/ Prefer not to answer** | | **Pro-life** | | **Total** | |
| --- | --- | --- | --- | --- | --- | --- | --- | --- | --- |
|  |  | N | % | N | % | N | % | N | % |
|  |  |  |  |  |  |  |  |  |  |
| **Total** |  | 358 | (100%) | 106 | (100%) | 208 | (100%) | 672 | (100%) |
|  |  |  |  |  |  |  |  |  |  |
| **Gender** | Man | 175 | 48.9% | 55 | 51.9% | 112 | 53.8% | 342 | 50.9% |
|  | Woman | 183 | 51.1% | 51 | 48.1% | 96 | 46.2% | 330 | 49.1% |
|  |  |  |  |  |  |  |  |  |  |
| **Age** | (mean) | 51.2 |  | 52.9 |  | 52.1 |  | 51.8 |  |
|  |  |  |  |  |  |  |  |  |  |
| **Race/ethnicity** | White | 240 | 67.0% | 55 | 51.9% | 149 | 71.6% | 444 | 66.1% |
|  | Latinx | 55 | 15.4% | 26 | 24.5% | 45 | 21.6% | 126 | 18.8% |
|  | Black/African American | 37 | 10.3% | 13 | 12.3% | 7 | 3.4% | 57 | 8.5% |
|  | Multiracial/Other | 26 | 7.3% | 12 | 11.3% | 7 | 3.4% | 45 | 6.7% |
|  |  |  |  |  |  |  |  |  |  |
| **Education** | High school or less | 91 | 25.4% | 49 | 46.2% | 80 | 38.5% | 220 | 32.7% |
|  | Some college | 95 | 26.5% | 27 | 25.5% | 53 | 25.5% | 175 | 26.0% |
|  | Bachelor or higher | 172 | 48.0% | 30 | 28.3% | 75 | 36.1% | 277 | 41.2% |
|  |  |  |  |  |  |  |  |  |  |
| **Church attendance** | Weekly/Monthly | 57 | 15.9% | 26 | 24.5% | 101 | 48.6% | 184 | 27.4% |
|  | Yearly | 133 | 37.2% | 51 | 48.1% | 65 | 31.3% | 249 | 37.1% |
|  | Never | 158 | 44.1% | 28 | 26.4% | 31 | 14.9% | 217 | 32.3% |
|  | (unknown) | 10 | 2.8% | 1 | 0.9% | 11 | 5.3% | 22 | 3.3% |
|  |  |  |  |  |  |  |  |  |  |
| **Party identification** | Republican | 46 | 12.8% | 27 | 25.5% | 98 | 47.1% | 171 | 25.4% |
|  | Democrat | 164 | 45.8% | 26 | 24.5% | 22 | 10.6% | 212 | 31.5% |
|  | Other/Any | 148 | 41.3% | 51 | 48.1% | 86 | 41.3% | 285 | 42.4% |
|  | (unknown) |  |  | 2 | 1.9% | 2 | 1.0% | 4 | 0.6% |
|  |  |  |  |  |  |  |  |  |  |
| **Urbanicity** | Rural | 58 | 16.2% | 19 | 17.9% | 48 | 23.1% | 125 | 18.6% |
|  | Urban | 116 | 32.4% | 36 | 34.0% | 70 | 33.7% | 222 | 33.0% |
|  | Suburban | 183 | 51.1% | 50 | 47.2% | 90 | 43.3% | 323 | 48.1% |
|  | (unknown) | 1 | 0.3% | 1 | 0.9% |  |  | 2 | 0.3% |
|  |  |  |  |  |  |  |  |  |  |
| **Region** | South | 119 | 33.2% | 49 | 46.2% | 84 | 40.4% | 252 | 37.5% |
|  | Northeast | 66 | 18.4% | 15 | 14.2% | 25 | 12.0% | 106 | 15.8% |
|  | Midwest | 78 | 21.8% | 19 | 17.9% | 42 | 20.2% | 139 | 20.7% |
|  | West | 95 | 26.5% | 23 | 21.7% | 57 | 27.4% | 175 | 26.0% |

**Table A2.** English and Spanish survey items

| English | Spanish |
| --- | --- |
| Awareness and agreement (Wave 2) | |
| *Roe v. Wade was the 1973 Supreme Court decision stating that a woman had a constitutional right to choose to have an abortion until she was about 24 weeks pregnant. Roe v. Wade was overturned (gotten rid of) in June 2022. The following questions will ask about Roe v. Wade and current abortion laws. Please answer them to the best of your ability.* | *Roe v. Wade fue la decisión de la Corte Suprema de 1973 que establecía que una mujer tenía el derecho constitucional a elegir tener un aborto hasta las aproximadamente 24 semanas de embarazo. Roe v. Wade fue revocado (eliminado) en junio de 2022. Las siguientes preguntas son sobre Roe v. Wade y las leyes sobre el aborto en la actualidad. Por favor, responda a las siguientes preguntas lo mejor que pueda.* |
| *Before taking this survey, had you heard that Roe v. Wade was overturned (gotten rid of) in June 2022?*  *1. Yes*  *2. No* | *Antes de responder a esta encuesta, ¿había escuchado usted que Roe v. Wade fue revocado (eliminado) en junio de 2022?*   1. *Sí* 2. *No* |
| *Do you agree or disagree with the decision to overturn (get rid of) Roe v. Wade?*  *1. Strongly agree*  *2. Agree*  *3. Unsure*  *4. Disagree*  *5. Strongly disagree* | *¿Está usted de acuerdo o en desacuerdo con la decisión de revocar (eliminar) Roe v. Wade?*  *1. Muy de acuerdo*  *2. De acuerdo*  *3. No estoy seguro(a)*  *4. En desacuerdo*  *5. Muy en desacuerdo* |
| Perceived measure of change in people’s abortion attitudes after *Dobbs* (Wave 2) | |
| *Have your opinions about the legality of abortion changed after Roe v. Wade was overturned (gotten rid of)?*  *1. Yes, now I am more supportive of abortion being legal.*  *2. Yes, now I am more opposed to abortion being legal.*  *3. No, my opinions on the legality of abortion have not changed.* | *¿Han cambiado sus opiniones sobre la legalidad del aborto desde que Roe v. Wade fue revocado (eliminado)?*  *1. Sí, ahora estoy más a favor de que el aborto sea legal.*  *2. Sí, ahora estoy más en contra de que el aborto sea legal.*  *3. No, mis opiniones sobre la legalidad del aborto no han cambiado.* |
| *[Open-ended item]*   - *Can you explain why you have become more supportive of abortion being legal after Roe v. Wade was overturned (gotten rid of)?* - *Can you explain why you have become more opposed to abortion being legal after Roe v. Wade was overturned (gotten rid of)?* | *[Open-ended item]*   - *¿Podría explicar por qué está más a favor de que el aborto sea legal desde que Roe v. Wade fue revocado (eliminado)?* - *¿Podría explicar por qué está más en contra de que el aborto sea legal desde que Roe v. Wade fue revocado (eliminado)?* |
| Measure of change in people’s abortion attitudes (Wave 1 and Wave 2) | |
| *Do you think abortion should be legal in all cases, legal in most cases, illegal in most cases, or illegal in all cases?*  *1. Legal in all cases*  *2. Legal in most cases*  *3. Illegal in most cases*  *4. Illegal in all cases* | *¿Cree que el aborto debería ser legal en todos los casos, legal en la mayoría de los casos, ilegal en la mayoría de los casos o ilegal en todos los casos?*   1. *Legal en todos los casos* 2. *Legal en la mayoría de los casos* 3. *Ilegal en la mayoría de los casos* 4. *Ilegal en todos los casos* |

**Table A3.** Open-ended codebook and Cohen’s kappa results for inter-rater reliability before consensus

| **Category** | **#** | **Code Name** | **Agreement** | **Expect Agreement** | **Cohen’s Kappa** | **Prob>0** |
| --- | --- | --- | --- | --- | --- | --- |
| **GREATER ENDORSEMENT** | | | |  |  |  |
| **A woman’s right** | 1 | Because it strengthened their belief that it is a woman’s decision | 95.52% | 50.18% | **0.9101** | 0.000 |
|  | 2 | Because they disagree with removing women’s right | 96.27% | 81.88% | **0.7941** | 0.000 |
|  | 3 | Because nobody should be forced to parent | 98.51% | 95.62% | **0.659** | 0.000 |
| **Against government involvement** | 4 | Because the government/men/people in power should not decide on women’s lives | 92.54% | 72.45% | **0.7291** | 0.000 |
|  | 5 | Because they don’t trust the government | 97.01% | 88.73% | **0.7352** | 0.000 |
|  | 6 | Because they think SCOTUS is politicized | 96.27% | 92.06% | 0.5302 | 0.000 |
| **Personal views on abortion** | 7 | Because the infinite reasons why women have abortions are neglected | 94.78% | 92.06% | 0.3422 | 0.000 |
|  | 8 | Because they disagree with gestational limits before viability | 98.51% | 97.06% | 0.4924 | 0.000 |
|  | 9 | Because they believe abortion should be legal at any time to prevent human suffering | 96.27% | 92.13% | 0.5262 | 0.000 |
|  | 10 | Because abortion should be allowed under certain circumstances | 95.52% | 84.92% | **0.7031** | 0.000 |
|  | 11 | Because abortion is healthcare | 96.27% | 88.09% | **0.6866** | 0.000 |
| **Consequences of *D v. J* Decision** | 12 | Because abortions will continue to happen, and they should be performed safely | 96.27% | 92.06% | 0.5302 | 0.000 |
|  | 13 | Because they took RvW for granted | 97.76% | 93.51% | **0.6552** | 0.000 |
|  | 14 | Because Dobbs raised awareness and they now believe more firmly in the importance of abortion | 92.54% | 83.69% | 0.5423 | 0.000 |
|  | 15 | Because it generated outrage against the decision | 89.55% | 84.75% | 0.3148 | 0.000 |
|  | 16 | Because they fear other fundamental rights may be at risk | 98.51% | 95.61% | **0.6599** | 0.000 |
|  | 17 | Because they or their family might be personally affected | 99.25% | 97.78% | **0.6633** | 0.000 |
|  | 18 | Because they feel outraged there are no exceptions | 97.76% | 92.13% | **0.7157** | 0.000 |
|  | 19 | Because the decision introduces inequality for women across the country | 95.52% | 88.67% | **0.6047** | 0.000 |
|  | 20 | Because the decision worsens the situation for vulnerable populations | 99.25% | 97.78% | **0.6633** | 0.000 |
|  | 21 | Because there is no protection or a welfare system to support families | 99.25% | 97.78% | **0.6633** | 0.000 |
|  | 22 | Because they disagree with criminalizing women and/or providers | 99.25% | 96.34% | **0.7964** | 0.000 |
| **Other** | 23 | I don’t know | 99.25% | 99.25% | 0 | . |
|  | 24 | Invalid/Incoherent | 97.76% | 96.34% | 0.3891 | 0.000 |

| **Category** | **#** | **Code Name** | **Agreement** | **Expect Agreement** | **Cohen's Kappa** | **Prob>0** |
| --- | --- | --- | --- | --- | --- | --- |
| **LOWER ENDORSEMENT** | | | |  |  |  |
| **Moral views against abortion** | 1 | Because they believe abortion is taking a life | 95% | 54.5% | **0.8901** | 0.000 |
|  | 2 | Because they want to protect the unborn’s right to live | 92.5% | 72.5% | **0.7273** | 0.000 |
|  | 3 | Because they believe life begins at conception | 97.5% | 92.75% | **0.6552** | 0.000 |
|  | 4 | Because they hold a strong general opposition to abortion | 97.5% | 88.25% | **0.7872** | 0.000 |
|  | 5 | Because abortion is morally wrong | 95% | 90.38% | 0.4805 | 0.000 |
|  | 6 | Because they believe it is God’s decision | 100% | 95.13% | **1** | 0.000 |
| **Judgement on women** | 7 | Because they want to avoid its use as birth control | 95% | 90.5% | 0.4737 | 0.001 |
|  | 8 | Because women should use contraceptive methods | 97.5% | 97.5% | 0 | 0.500 |
|  | 9 | Because they want to protect women from suffering after abortion | 100% | 95.13% | **1** | 0.000 |
|  | 10 | Because they want to make people responsible for their acts | 100% | 86.13% | **1** | 0.000 |
| **Legal views about abortion** | 11 | Because states would legislate better than the federal government | 95% | 90.38% | 0.4805 | 0.000 |
|  | 12 | Because they want to see more regulations in place | 97.5% | 88.25% | **0.7872** | 0.000 |
|  | 13 | Because they don’t believe in gestational limits or viability | 100% | 95.13% | **1** | 0.000 |
|  | 14 | Because they support some circumstances, but they are generally against it. | 100% | 95.13% | **1** | 0.000 |
| **Other** | 16 | I don’t know | 0% | 95.13% | **1** | 0.000 |
|  | 17 | Invalid/Incoherent | 97.5% | 88.25% | **0.7872** | 0.000 |

**Table A4.** Multinomial regression models of changes in attitudes after *Dobbs v. Jackson*

|  | **Lower Endorsement vs.  No change** | | | |  | **Greater Endorsement vs.  No change** | | | |
| --- | --- | --- | --- | --- | --- | --- | --- | --- | --- |
|  | Model 1 | | Model 2 | |  | Model 3 | | Model 4 | |
|  | *Perceived change* | | *Response change across waves* | |  | *Perceived change* | | *Response change across waves* | |
|  | RRR | P>\|z\| | RRR | P>\|z\| |  | RRR | P>\|z\| | RRR | P>\|z\| |
| **Abortion identity (Ref. Pro-life)** |  |  |  |  |  |  |  |  |  |
| Pro-choice | 0.14 | 0.001 | 2.67 | 0.009 |  | 9.65 | 0.000 | 0.83 | 0.529 |
| Both/Neither/Prefer not to answer | 0.21 | 0.016 | 3.26 | 0.006 |  | 3.23 | 0.015 | 2.00 | 0.035 |
| **Gender (Ref. Man)** |  |  |  |  |  |  |  |  |  |
| Woman | 0.70 | 0.362 | 1.00 | 0.994 |  | 0.73 | 0.129 | 1.44 | 0.096 |
| **Age** | 1.02 | 0.107 | 1.00 | 0.891 |  | 1.00 | 0.794 | 1.00 | 0.542 |
| **Race/ethnicity (Ref. White)** |  |  |  |  |  |  |  |  |  |
| Latinx | 9.27 | 0.000 | 1.51 | 0.277 |  | 1.59 | 0.131 | 1.62 | 0.129 |
| Black/African American | 3.00 | 0.196 | 1.56 | 0.298 |  | 1.14 | 0.693 | 1.60 | 0.201 |
| Multiracial/Other | 2.62 | 0.220 | 0.62 | 0.386 |  | 1.15 | 0.722 | 0.58 | 0.250 |
| **Education (Ref. HS or less)** |  |  |  |  |  |  |  |  |  |
| Some college | 0.95 | 0.921 | 0.66 | 0.210 |  | 0.77 | 0.334 | 0.85 | 0.542 |
| Bachelor or higher | 0.88 | 0.802 | 0.53 | 0.055 |  | 0.84 | 0.498 | 0.75 | 0.298 |
| **Church attendance (Ref. Weekly/Monthly)** |  |  |  |  |  |  |  |  |  |
| Yearly | 0.90 | 0.808 | 0.63 | 0.155 |  | 1.52 | 0.174 | 0.88 | 0.639 |
| Never | 0.64 | 0.409 | 0.33 | 0.004 |  | 1.53 | 0.186 | 0.94 | 0.855 |
| **Party identification (Ref. Republican)** |  |  |  |  |  |  |  |  |  |
| Democrat | 0.40 | 0.149 | 0.49 | 0.074 |  | 1.37 | 0.335 | 0.68 | 0.260 |
| Other/Any | 0.81 | 0.651 | 0.84 | 0.591 |  | 1.18 | 0.600 | 0.99 | 0.984 |
| **Urbanicity (Ref. Rural)** |  |  |  |  |  |  |  |  |  |
| Urban | 0.89 | 0.841 | 1.18 | 0.676 |  | 1.27 | 0.497 | 1.43 | 0.274 |
| Suburban | 0.79 | 0.665 | 0.83 | 0.611 |  | 2.02 | 0.022 | 1.03 | 0.930 |
| **Region (Ref. South)** |  |  |  |  |  |  |  |  |  |
| Northeast | 2.77 | 0.109 | 0.41 | 0.055 |  | 0.68 | 0.206 | 0.75 | 0.397 |
| Midwest | 3.98 | 0.012 | 0.96 | 0.913 |  | 0.91 | 0.730 | 1.41 | 0.225 |
| West | 2.11 | 0.176 | 1.38 | 0.359 |  | 0.50 | 0.022 | 1.11 | 0.732 |
|  |  |  |  |  |  |  |  |  |  |
| Constant | 0.01 | 0.000 | 0.25 | 0.019 |  | 0.33 | 0.063 | 0.19 | 0.002 |
| Number of observations | 642 |  | 626 |  |  | 642 |  | 626 |  |

**Figure A1.** Participants’ change in attitudes toward abortion legality by identification with abortion identity labels

*Do you think abortion should be legal in all cases, legal in most cases, illegal in most cases, or illegal in all cases?*

***Panel A: Pro-choice Panel B: Both/Neither/Prefer not to answer***


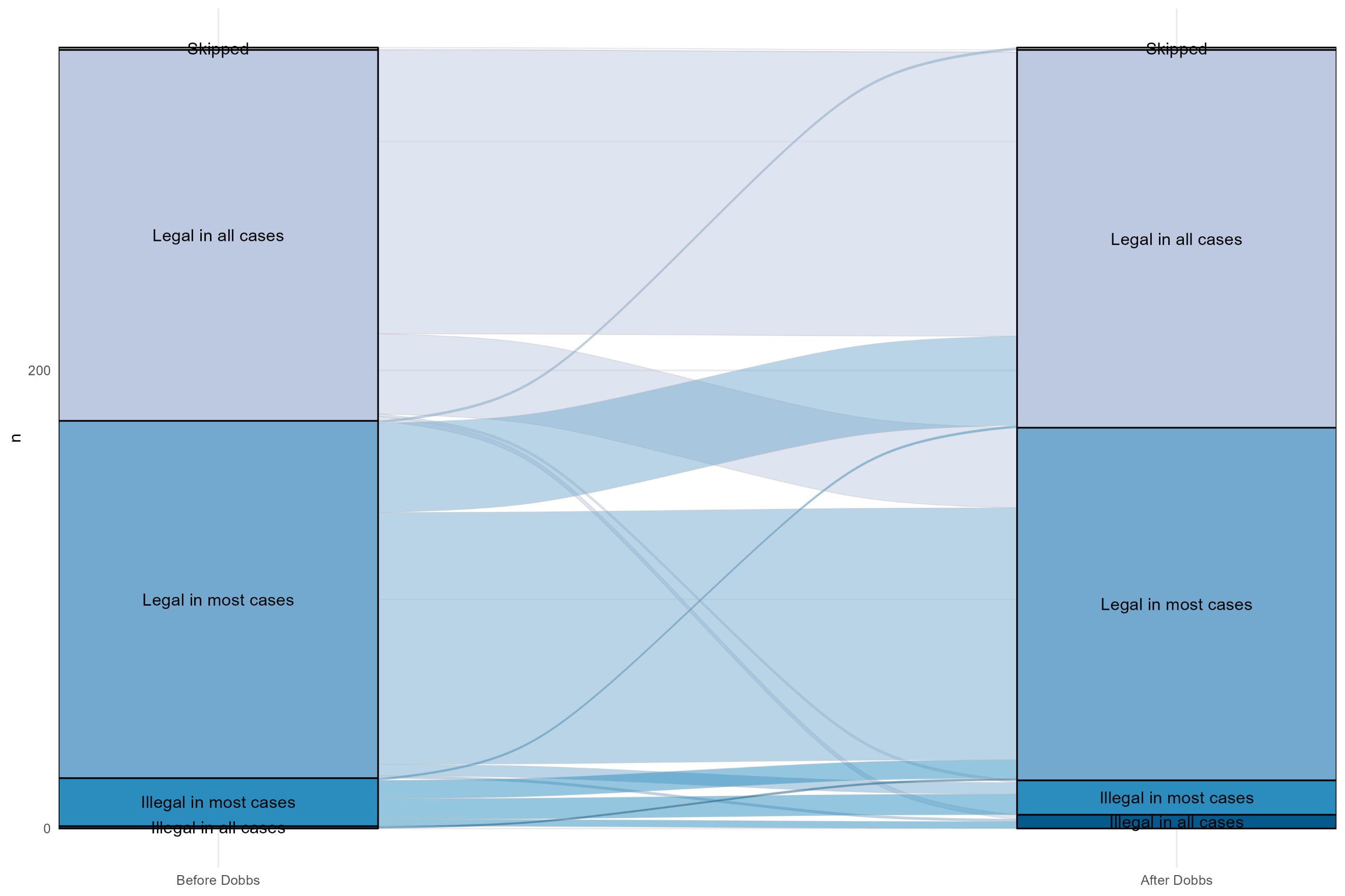

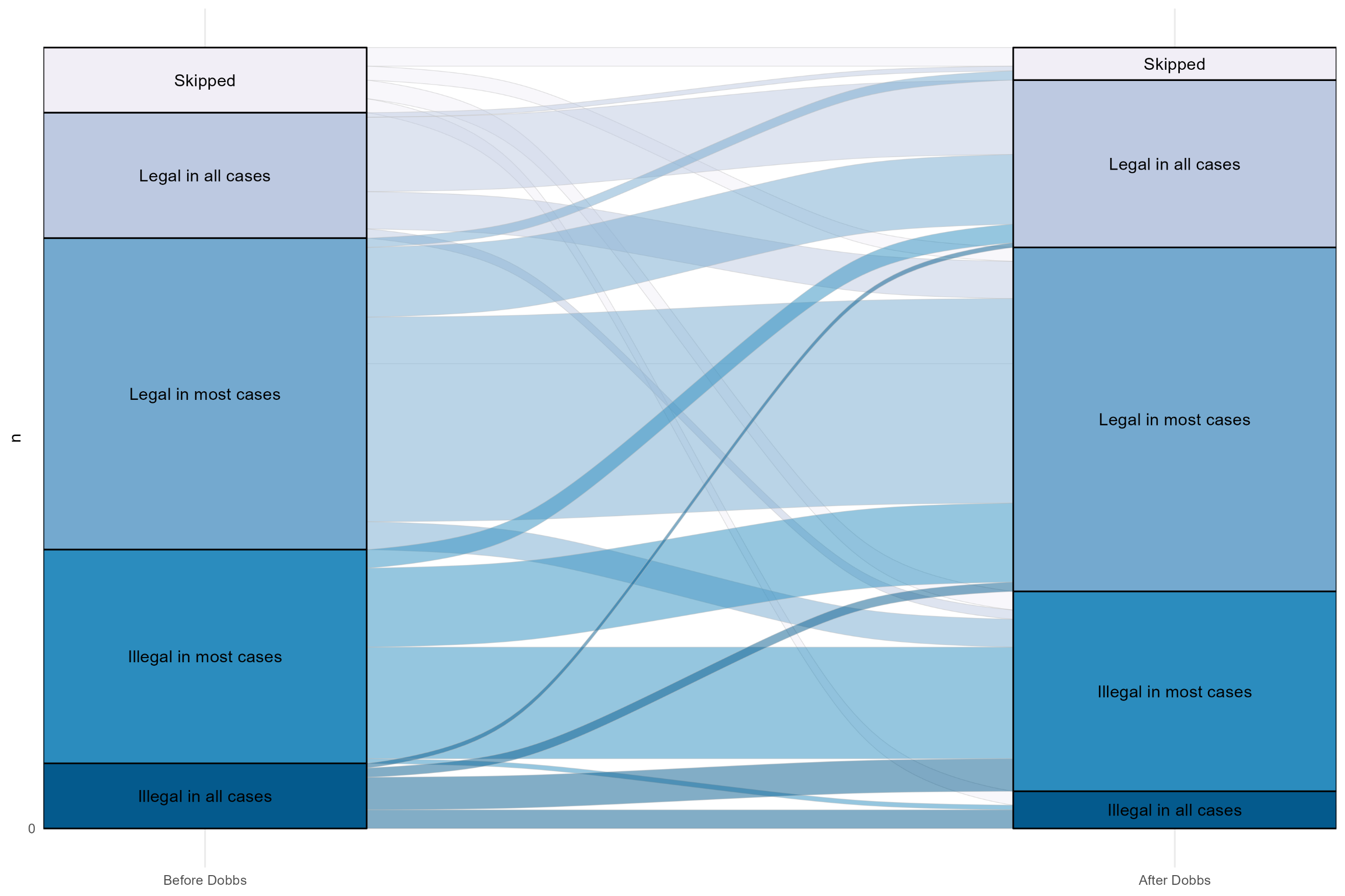


***Panel C: Pro-life Panel D: Total***


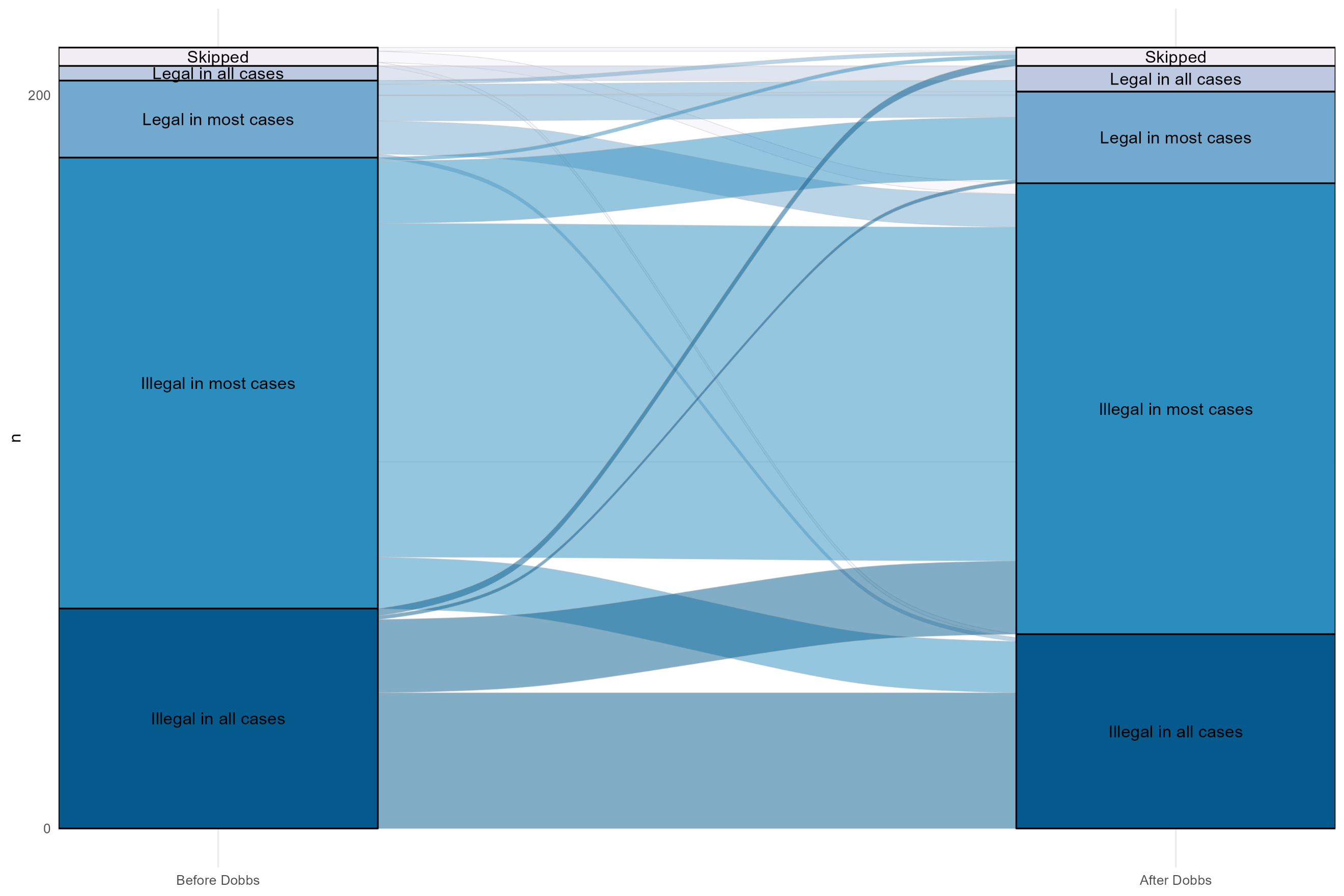

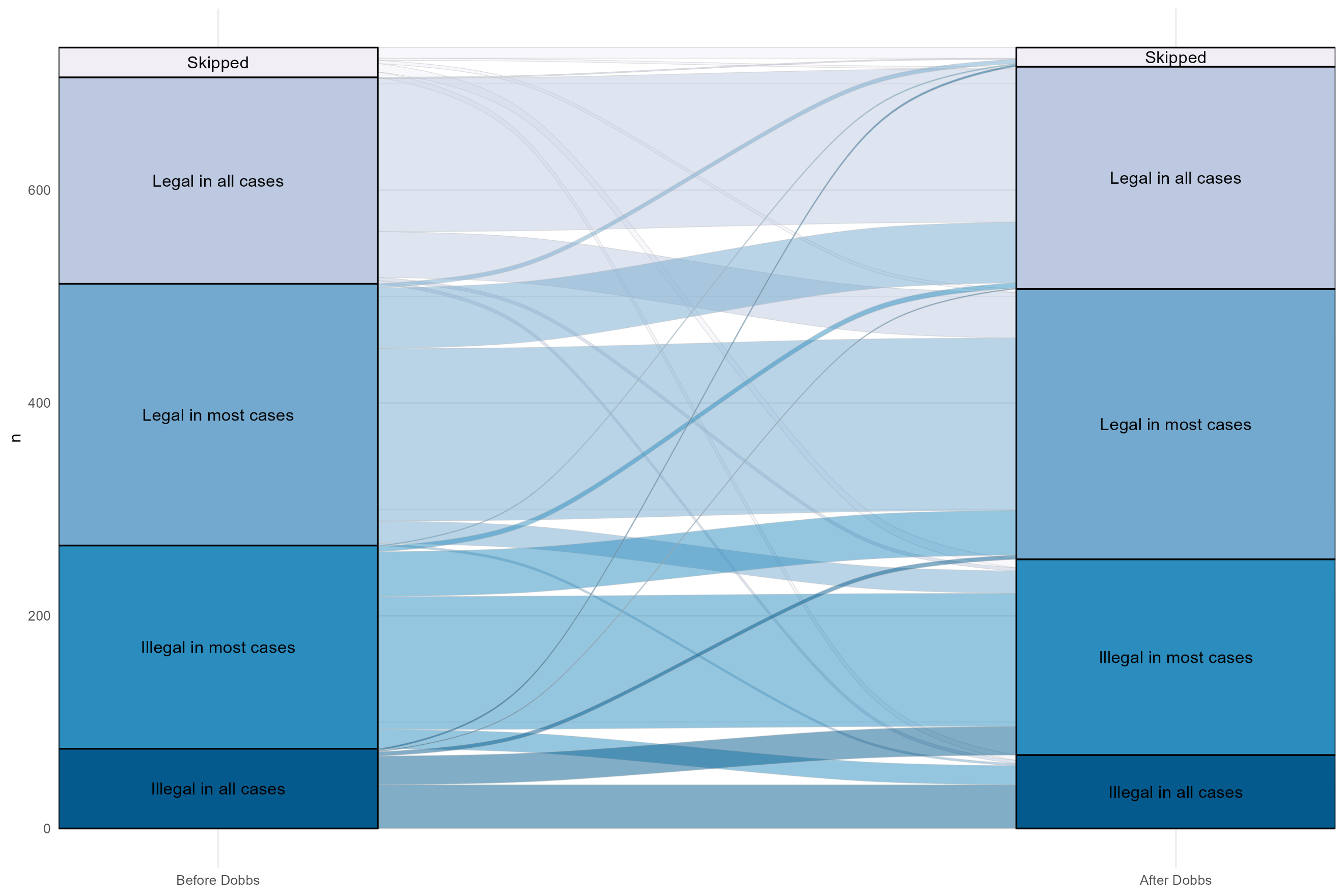

Supplement: Appendix [file ZRHM_A_2518669_SM7264.docx]
